# Endoscopic Features of Autoimmune Gastritis: Focus on Typical Images and Early Images

**DOI:** 10.3390/jcm11123523

**Published:** 2022-06-19

**Authors:** Maiko Kishino, Kouichi Nonaka

**Affiliations:** Department of Digestive Endoscopy, Tokyo Women’s Medical University, Tokyo 162-8666, Japan; kishino.ige@twmu.ac.jp

**Keywords:** autoimmune gastritis (AIG), image-enhanced endoscopy (IEE), early stage

## Abstract

Autoimmune gastritis (AIG) is chronic atrophic gastritis caused by an autoimmune mechanism of unknown etiology and presents with various pathological conditions by causing an achlorhydria state through parietal cell damage. The most characteristic endoscopic finding in AIG is advanced corpus-dominant mucosal atrophy. A recent study that examined several cases in Japan revealed the presence of endoscopic features other than corpus-dominant advanced atrophy. Remnants of oxyntic mucosa and sticky adherent dense mucus were found in ≥30% of cases, and hyperplastic polyps were found in ≥20% of cases. In image-enhanced endoscopy (IEE), white globe appearance (WGA) was observed in 32% of AIG cases. Additionally, some reports have stated that the findings in AIG cases using IEE showed cast-off skin appearance (CSA) and foveola type mucosa; however, a consensus is yet to be achieved. These endoscopic results were found in cases of advanced-stage AIG. There have been few reports concerning early-stage AIG cases. In these few reports, all of the cases were pathologically diagnosed as early AIG. In all of the cases, the pathological findings almost always showed neither parietal cell destruction nor atrophy. Endoscopic findings such as “mosaic pattern with slight swelling of the areae gastricae”, “diffuse reddened and edematous gastric fundic gland mucosa”, and “pseudopolyp-like nodules” may be common characteristics of early images. In such early cases, high antibody titers, no atrophic changes, and few clinical abnormal findings were shown. Endoscopists are expected to update their knowledge regarding AIG diagnosis with the evolution of imaging equipment.

## 1. Introduction

The definition of autoimmune gastritis (AIG) was clarified in a study by Strickland and Mackay [1]. They classified chronic atrophic gastritis into two types, type A and type B, depending on the presence or absence of autoantibodies. Type A gastritis was characterized by the presence of autoantibodies and mucosal atrophy of the corpus glandular region and hypergastrinemia, while Type B gastritis did not involve autoantibodies and mainly occurred in the prepyloric region. Later, Kurokawa et al. reported atrophic gastritis that was predominant in the corpus glandular region, which is characteristic of type A gastritis, as “endoscopic reversed atrophic type gastritis” [2]. The term type A gastritis has been used as a synonym for AIG. However, there have been reports of cases in which autoantibodies were present but there was no evidence of chronic atrophic gastritis in early images. In other words, these cases of autoimmune gastritis had characteristics that were different from those of conventional type A gastritis. Therefore, the term “AIG” is now considered to be more appropriate [3].

AIG is a disease in which anti-parietal-cell antibodies destroy parietal cells through an unexplained autoimmune mechanism. Hypergastric acid secretion is reduced to an achlorhydria state due to the destruction of parietal cells, causing hypergastrinemia. Intrinsic factor secretion is also reduced, and the appearance of anti-intrinsic factor antibodies causes the impaired absorption of vitamin B12 [4]. Clinically, AIG manifests as a variety of systemic medical conditions, such as iron deficiency anemia, pernicious anemia, spinal cord nerve disease, gastric tumors, and complications with other autoimmune diseases [5,6,7].

The use of endoscopy plays an important role in the diagnosis of AIG. Recently, in Japan, in a multicenter study that was composed of about 200 cases, Terao et al. reported some characteristic findings in AIG that differ from the previously known “endoscopic reversed atrophic type gastritis” [8]. In addition to these findings, this report also described a recent review from Japan that discussed early AIG cases that were examined prior to the onset of atrophic gastritis and studies referring to mucosal findings in AIG using IEE. A characteristic of early AIG is mild lymphocyte infiltration in the middle-to-deep layers of the lamina propria mucosa, but neither marked parietal cell destruction nor atrophy have been shown in histopathological findings. This review may contribute to the correct diagnosis of gastric mucosal findings that were previously misdiagnosed as “non-specific redness” in early AIG images. In this review, we also suggest that IEE could be used to indicate differences in the causes of atrophic gastritis that present as differences in endoscopic findings. In other words, IEE findings may lead to the clarification of the pathophysiology of gastrointestinal mucosa.

The present review will deepen the knowledge of early AIG, which is quite different from typical AIG. Increasing the diagnostic rate of early AIG will enable the early initiation of surveillance for the prevention of vitamin B12 deficiency and iron deficiency and the detection of gastric neoplastic lesions.

At this time, no aggressive treatment for AIG has been established. However, if the accumulation of these findings reveals the natural history of AIG, the active verification of radical treatment may be required. High-resolution images may show atypical findings that were previously unknown. Based on such information, we summarized the findings from AIG diagnoses that endoscopists should use to update their knowledge based on reports of IEE findings and early images of AIG cases.

## 2. Pathophysiology

Autoimmune gastritis is a form of chronic gastritis in which an unexplained autoimmune mechanism causes the chronic inflammation of the mucosa of the fundic gland region, leading to severe mucosal atrophy. Toh et al. revealed that the target antigen of the autoimmune mechanism is H+/K+ ATPase, and they believed anti-parietal-cell antibodies and anti-intrinsic-factor antibodies to be involved in a series of mechanisms [9]. Meanwhile, from an analysis of disease model mice, Kojima and Nishizuka et al. suggested that autoreactive CD4+ T cells target H+/K+ ATPase and that this plays a central role in the onset and pathogenesis of AIG. Furthermore, Kojima and Nishizuka et al. stated that anti-parietal-cell antibodies were produced in the process of parietal cell destruction and that they have low pathological significance [10].

Furthermore, some reports have shown that *Helicobacter pylori* (*Hp.*) infection plays a role in the development of AIG. These studies showed that the mechanism of gastric mucosal damage caused by *Hp.* infection involves the production of autoantibodies against H+/K+ ATPase and that the resulting parietal cell damage progresses to AIG [11,12]. However, there was a report of cases in which AIG progressed rapidly due to *Hp.* eradication [13], and another report showed that AIG mucosal damage disappeared when AIG mice were infected with *Hp.* in mouse experiments [14]. However, a consensus has not yet been reached regarding this point.

## 3. Epidemiological and Clinical Findings

AIG was previously thought to be predominant in older women in Scandinavia, but with a recent increase in the number of reports, it is now believed that there are no racial or age differences in the prevalence of AIG [15]. In terms of sex differences, AIG is more common in female patients [16]. It has been thought that there are fewer AIG cases in Japan than in Western countries. However, a study in a health-check facility in Japan reported that 0.89% of the individuals examined (0.6% of male and 1.12% of female participants) had AIG. Furthermore, the authors of that report stated that the actual prevalence of AIG may be even higher given the presence of early cases and cases in which endoscopic diagnosis was difficult due to *Hp.* infection [17].

In AIG, severe parietal cell damage causes the impaired absorption of iron and vitamin B12, which may be accompanied by iron deficiency anemia, pernicious anemia, and malignant associative spinal cord degeneration [4,5,6,7].

In addition, AIG is often associated with other autoimmune diseases, such as autoimmune thyroiditis, Sjogren’s syndrome, and type 1 diabetes [15], and it is therefore considered a systemic disease. In particular, complications with autoimmune thyroiditis are known and are classified in the 3B category of polyglandular autoimmune syndrome [18].

## 4. Findings of typical AIG

### 4.1. Histopathological Findings

**Figure 1:**
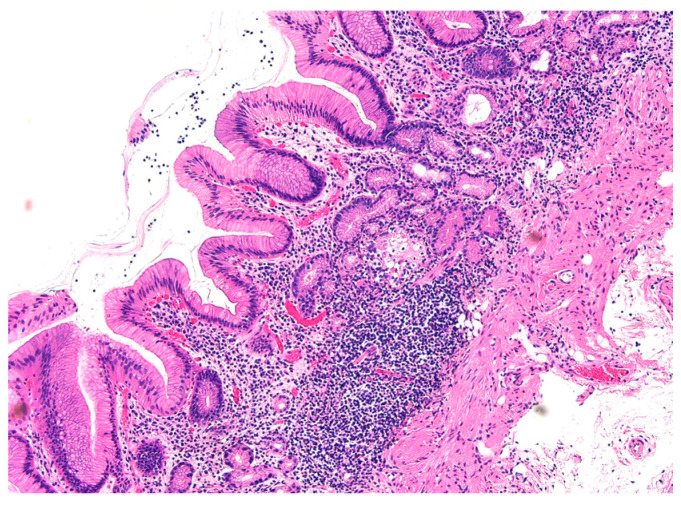
Histopathological image of AIG. Lymphocytic infiltration is observed in the deep layer of the lamina propria. The parietal cells in the lamina propria almost disappear. On the other hand, the density of the glandular epithelium is relatively maintained.

The histopathological imaging of AIG typically shows a marked decrease in lymphocytes, eosinophil infiltration, and parietal cells centered on the deep mucosa of the fundic glands. Lymphocytes infiltrate while individually destroying fundic gland cells, including parietal cells and chief cells. However, no inflammation of the glandular epithelium is observed. As the fundic gland cells of the lamina propria decrease in number, the thickness of the lamina propria becomes less than that of the mucosal epithelial layer (Figure 1).

In the pathological diagnosis of AIG, the stage is classified on the basis of the respective findings [19]. The stages are divided into three phases: early phase, florid phase, and end stage. In the early phase, lymphocyte infiltration and plasma cell infiltration are diffuse or nest-like in the deep layer of the mucosa. In the florid phase, lymphocyte infiltration becomes severe, the fundic glands disappear, and a high degree of atrophy is observed. In the end stage, changes in the florid phase progress, and the fundic glands mostly disappear. With the disappearance of the target of the autoimmune reaction, inflammation subsides, and the infiltration of inflammatory cells disappears. When fundic gland cells begin to disappear, mucus cell supplementation occurs, and pseudopyloric gland metaplasia appears. Upon the disappearance of the fundic glands, they are replaced by true pyloric metaplasia, intestinal metaplasia, and pancreatic acinar metaplasia. In addition, parietal cell damage causes the trophic effect of gastrin, enterochromaffin-like (ECL) cells proliferate, and a histological image called ECL cell hyperplasia appears. The histological image of ECL cell hyperplasia also changes from linear to tubular and gradually to nodular as inflammation progresses. The nodular hyperplasia of ECL cells is synonymous with endocrine cell micronests (ECMs), and the nodular proliferation of ECL cells with a maximum size of ≥0.5 mm is considered neoplastic [19,20]. The main site of these inflammations is the lamina propria. The crypt epithelium is relatively preserved during the active phase of inflammation, resulting in a high crypt epithelium/intrinsic gland ratio. This is considered to be one of the distinguishing points in the histological image of chronic gastritis due to *Hp* infection, in which inflammation begins in the glandular epithelium.

### 4.2. Endoscopic Findings

#### 4.2.1. Findings via White Light Observation

**Figure 2:**
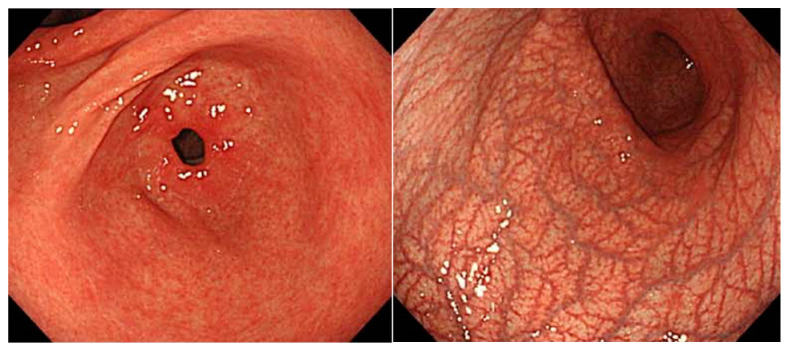
Image of endoscopic reversed atrophic type gastritis.

A well-known endoscopic finding in AIG is the presence of so-called “reverse atrophy” (Figure 2), or severe atrophic gastritis, which predominantly occurs in the glandular region of the gastric corpus. Atrophic gastritis predominant in the gastric corpus was reported in the late 19th and early 20th centuries in studies of cases of pernicious anemia. Kurokawa et al. reported that 85% of patients with pernicious anemia had widespread atrophic gastritis predominantly in the gastric corpus area and termed it “endoscopic reversed atrophic type gastritis” [2]. The authors described the finding as follows: “the mucosa of the whole gastric body was severely atrophic, in contrast to the less severely atrophic antrum”. This finding is well-known to many endoscopists as a key finding that triggers the diagnosis of AIG.

**Figure 3:**
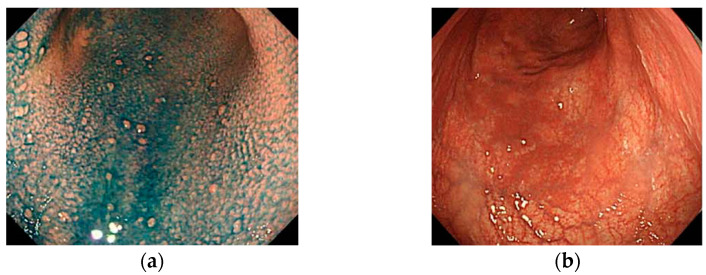
Images of remnants of oxyntic mucosa: (**a**) pseudopolyp-like type; (**b**) extensive type.

A characteristic finding, as well as “endoscopic reversed atrophic type gastritis”, is the presence of remnants of oxyntic mucosa (Figure 3). AIG forms atrophic gastritis over a wide area of the corpus gland but may be accompanied by some remaining normal fundic glandular mucosa. Krasinskas et al. reported multiple polyps consisting of gastric-acid-secreting tissue that appear in the atrophic mucosa of patients with AIG as oxyntic mucosa pseudopolyps [21]. This finding is consistent with the presence of remnants of oxyntic mucosa, as reported by Terao et al., and is considered particularly consistent with pseudopolyp type mucosa, the morphology of which Terao et al. classified in detail. Terao et al. reported that “remnants of oxyntic mucosa” were found in 31.5% of cases [8].

**Figure 4:**
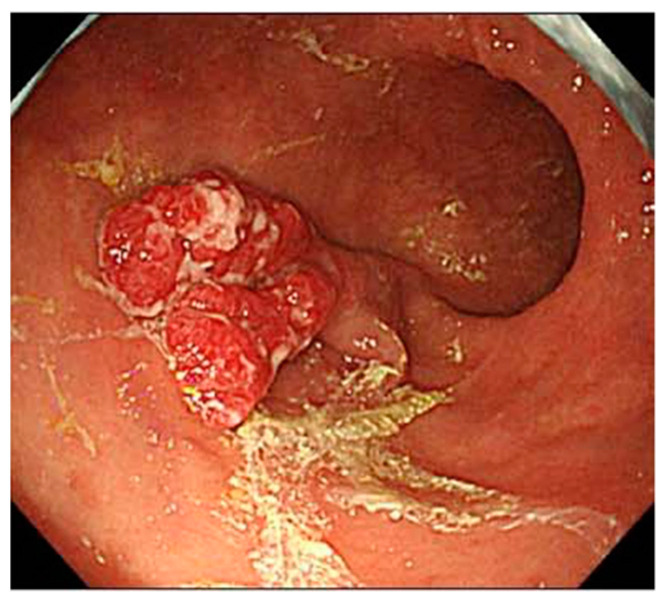
Image of hyperplastic polyp.

The examination of a large number of cases has shown that the major endoscopic feature in AIG is the frequent occurrence of hyperplastic polyps (Figure 4) [22,23,24]. Reports have stated that multiple polyps develop in the gastric corpus and appear in the end stage of AIG. In addition, Yaita et al. reported that the coexistence rate of hyperplastic polyps was higher in AIG cases with gastric cancer than in those without gastric cancer, and Zhang et al. reported that polyps were relatively large, with several polyps appearing at the same time [25,26]. In the study by Terao et al., they reported that gastric hyperplastic polyps were found in 21.2% of AIG cases [8].

**Figure 5:**
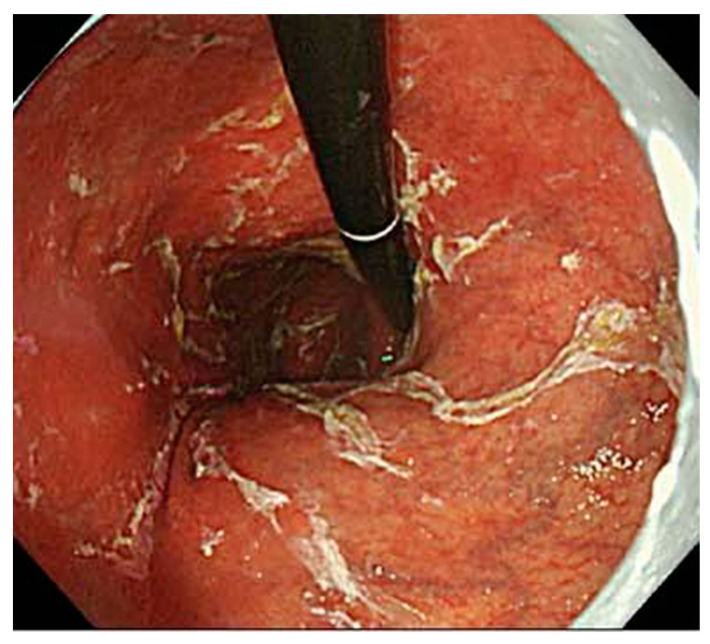
Images of sticky adherent dense mucus.

The newest endoscopic finding in AIG cases is sticky adherent dense mucus (Figure 5). Sticky adherent dense mucus refers to mucus that adheres from the fundus to the upper body of the stomach, is pale yellow to white, and cannot be easily removed with water. Terao et al. explained this finding in a previous paper, reporting that sticky adherent dense mucus was found in 32.4% of the total number of cases [8]. Furuta et al. described the relationship between the development of sticky adherent dense mucus and the growth of urease-producing bacteria other than *Hp.* in the stomach in an achlorhydria state. In addition, it was reported that the presence of bacterium caused the misdiagnosis of *Hp.* infection via urea breath tests, leading to the risk of being refractory to eradication therapy [27]. Therefore, the test results of *Hp.*-negative AIG cases were false positive, and cases were misdiagnosed as unfit for eradication. The presence of sticky adherent dense mucus is an important clinical problem associated with AIG.

AIG is often overlooked. In our single-center study concerning AIG endoscopy, AIG was missed in previous endoscopies in 49% of the patients. When the relationship between the presence or absence of this oversight and endoscopic findings was investigated, associations were found regarding redness, erosion, intestinal metaplasia in the antrum, and xantoma in the gastric body. Based on this result, the presence of findings in the antrum (i.e., in cases with abnormalities in their mucosa) may cause AIG to be undiagnosed and missed in spite of the presence of severe atrophy that spreads toward the gastric corpus [28].

**Figure 6:**
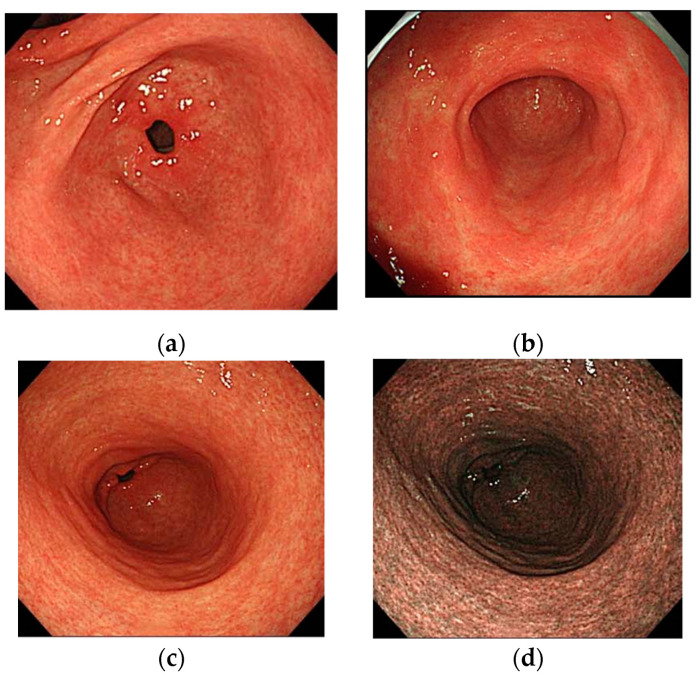
Images of antral mucosa with AIG: (**a**) normal mucosa; (**b**) patchy redness; (**c**,**d**) circular wrinkle-like pattern (white light image/NBI image).

Regarding findings in the antrum in AIG cases, Maruyama et al. reported that in approximately 30% of cases, the pyloric gland area was confined to the area around the pyloric ring. This means that the atrophied gastric corpus extends to the pyloric gland area [29]. Terao et al. also examined and reported mucosal findings relating to the pyloric gland area of AIG in detail [8]. According to their report, “patchy redness” was observed in 22.1% of the cases, “circular wrinkle-like pattern” in 22.1% of the cases, “red streak” in 10.4% of the cases, and “raised erosion” in 3.6% of the cases. Less than half of all of the patients (or 40%) were deemed to have relatively normal mucosa when these findings were not considered. Findings in the antrum in AIG cases indicate the existence of several variations of mucosa as opposed to normal mucosa alone (Figure 6).

**Figure 7:**
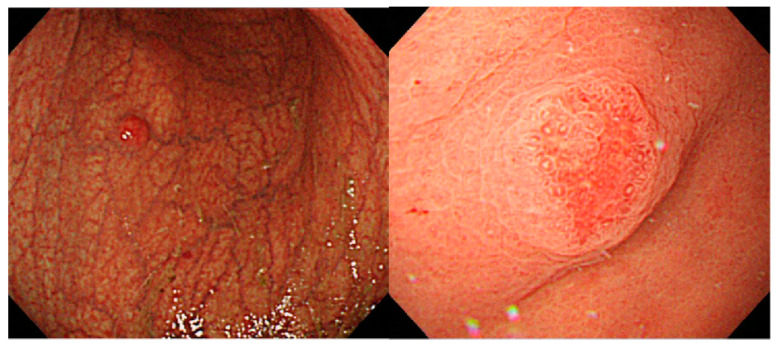
Images of neuroendocrine tumors (NETs) with AIG.

**Figure 8:**
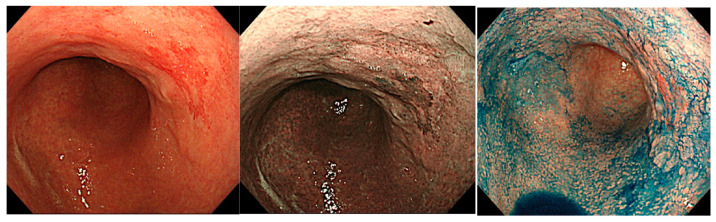
Images of gastric cancer with AIG.

Tumorous lesions associated with AIG include neuroendocrine tumors (NETs) and gastric cancer (Figure 7 and Figure 8). The combination of these two lesions has long been established. Prolonged hypergastrinemia causes an increase in enterochromatin-like cells (ECL cells) in the fundic gland region, leading to the formation of endocrine cell micronests (ECMs), resulting in the development of NETs. Furthermore, gastric cancer occurs due to intestinal metaplasia which occurs as a result of a high degree of atrophy of the mucosa, caused by a high degree of damage to fundic gland cells. Vannella et al. reported that the incidence of gastric cancer in pernicious anemia was 0.27% per year and that the estimated relative risk of gastric cancer in patients with pernicious anemia was nearly seven times higher [30]. Kamada et al. provided the complication rates for NETs and gastric cancer in patients with AIG or pernicious anemia for each existing worldwide report [31]. According to their report, the complication rate for NETs was 2.4 to 15.4%, and that for gastric cancer was 0.7 to 12.2%.

#### 4.2.2. Findings via IEE

**Figure 9:**
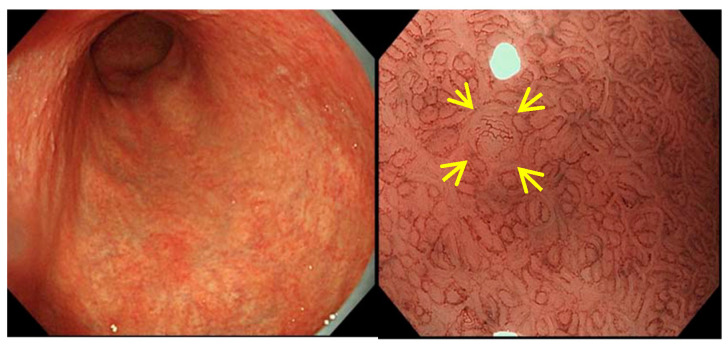
Images of white globe appearance with AIG.

Regarding the characteristic findings from magnifying endoscopy in AIG, the aforementioned report by Terao et al. stated that white globe appearance (WGA) (Figure 9) was observed in the lesser curvature of the gastric corpus in 32% of cases [8]. As reported by Doyama et al., WGA is present in the margin of differentiated gastric cancer [32], but it has also been shown to be relatively frequent in AIG as well.

**Figure 10:**
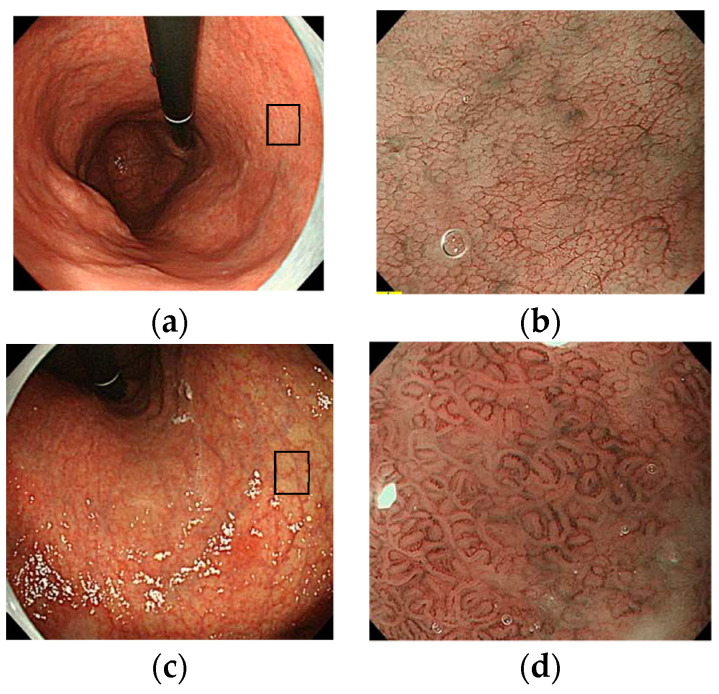
Magnifying NBI images of corpus mucosa with AIG. (**a**,**b**) Foveolar type; (**c**,**d**) groove type. The rates of foveolar type and groove type were 40% (17/43) and 37% (15/43), respectively, in our study [29].

There are few, albeit interesting, reports regarding IEE findings in the corpus mucosa in AIG. Yagi et al. described the characteristic IEE finding for AIG as “a densely arranged image of slightly larger circular or oval shaped pits” [33]. However, Maruyama et al. reported that they found “an image of a network of preserved capillaries but without a central glandular opening” in 59% of AIG cases [34]. This finding was labeled the “cast-off skin appearance” (CSA) [31,34]. Kato et al. reported a comparison of IEE images of AIG, current *Hp*-infected cases, and non-*Hp*-infected cases [35]. They showed different IEE images of AIG and *Hp* gastritis with almost equally moderate or greater atrophy. The IEE findings in AIG revealed that all of the cases of AIG included the presence of extensive “foveola type” mucosa, which Kanzaki et al. deemed to be a finding obtained from NBI magnifying endoscopy in chronic atrophic gastritis in the gastric corpus [36]. On the other hand, the same “foveola type” mucosa was observed in <10% of *Hp* gastritis cases, and most cases had “groove type” mucosa. However, Kato et al. stated that none of the pathological findings in the target AIG cases showed intestinal metaplasia, and therefore, staging bias may have affected the outcome. In a study at our facility, NBI magnifying endoscopic findings in the lesser curvature of the middle body, which is a typical example of AIG with predominantly severe atrophy in the corpus gland area, were examined. Out of 43 cases, 17 (40%) had extensive findings equivalent to “foveola type” mucosa, wherein a network structure of epithelialized blood vessels was preserved, and 15 cases (34%) had “groove type” mucosa, which was shown to have wide tubular structures (Figure 10) [28].

The center of AIG inflammation is the lamina propria of the gastric wall. Therefore, the mucosal surface layer is not inflamed in the early or the active stages. We believe that these findings manifest as “CSA” and “foveola type” mucosa. This is different from *Hp*-infected gastritis, wherein inflammation begins in the glandular epithelium.

However, it is possible that findings differ depending on the stage of AIG, and it is difficult to narrow down the characteristic findings from IEE. Hence, the further accumulation of IEE findings based on stage assessment is needed.

## 5. Findings of Early AIG

**Figure 11:**
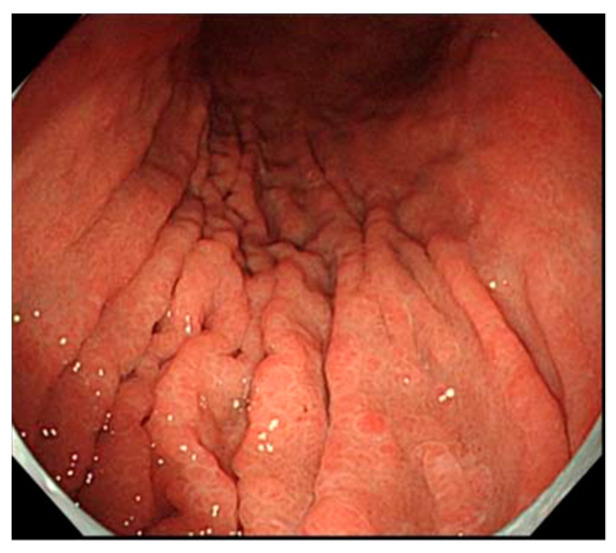
The findings of a case diagnosed as early AIG. It presents a mosaic pattern consisting of swollen gastric areas in the corpus.

The endoscopic image of AIG shown above (Figure 2) is a typical image of the gastric mucosa, wherein inflammation has progressed to a certain extent. There have been only a few reports containing endoscopic images in early AIG, although several reports have recently been published. There are reports of preatrophic gastritis AIG cases that do not display atrophy of the corpus glandular region that are known to many endoscopists. In one case reported by Ayaki et al. [37], endoscopy performed for screening showed a mosaic pattern with slight swelling of the areae gastricae restricted to the corpus; however, normal mucosa was seen in the prepyloric region. In our case, the endoscopic findings showed normal gastric pyloric gland mucosa, as well as diffuse reddened and edematous gastric fundic gland mucosa. The detailed observation of the reddish mucosa revealed reddened and edematous changes in the gastric areas as mixed findings in the small-ridge and depressed pale areas [38]. We also presented an IEE image of an early AIG case, and according to the IEE observation of the mucosa of the gastric corpus, the structure was maintained, showing a regular coil-shaped SECN pattern and a regular curved MCE pattern. It was determined that the AIG image before atrophy was clearly captured. Our finding was similar to the mosaic pattern described by Ayaki et al. In both reports, histopathological biopsy examination from the greater curvature of the gastric corpus showed the local deep mucosal infiltration of lymphocytes and plasma cells, but a normal lamina propria remained. Immunostaining showed only fundic gland cell damage and linear ECL cell hyperplasia in areas with inflammatory cell infiltration. PCA showed a high titer, and the serum gastrin level showed a slight increase. The mosaic pattern of the gastric-area swelling in the corpus suggests that this may be a characteristic of early AIG before the appearance of atrophy (Figure 11). In the cases reported by Kotera et al. [39], who reported endoscopic findings from AIG cases in the earliest stage, a slightly reddish nodule resembling a pseudopolyp was noted. Such a nodule is one of the characteristic findings of AIG, typical of the curvatures of the gastric corpus without atrophy. In both cases, atrophic gastritis in the gastric corpus gland area became apparent over time, and the presence of reddish nodules decreased. Typical extensive severe atrophic gastritis was not observed using endoscopy. Kotera et al. recently reported an early case of AIG that could only be described as presenting completely normal mucosa [40]. In that case, steroid treatment for interstitial pneumonia was performed for a period of time, but PCA increased, and IFA became positive during the subsequent course. However, the endoscopic image did not show any atrophy, and the mucosa could be considered almost normal. Pathological examination revealed normal-looking mucosa in the greater curvature that looked like early AIG, with characteristics such as mild atrophy and lymphocyte infiltration in the deep mucosa, the local disappearance of fundic gland cells in the lamina propria, and the linear hyperplasia of ECL cells.

Table 1 shows the endoscopic findings, pathological findings, autoantibodies, and serum markers of the early AIG cases according to the four reports discussed above. From these four reports, it can be determined multiple pseudopolyps in the corpus glandular region, diffuseness of the swollen gastric areas, and mosaic-like spread may be findings present in early images. In all of the cases, the antibody titer of PCA was high. Compared with typical images, the gastrin level was low and the PG1 level was high.

Only a few reports of early AIG exist, and the characteristics of its endoscopic findings have not yet been clarified. In the future, in order to increase the correct diagnosis of early cases, it is necessary to try to confirm autoantibodies and comorbidities, such as other autoimmune diseases at the time of endoscopy, using the previously reported features.

## 6. Conclusions

In Japan, the number of people infected with *Hp* is clearly decreasing, and the incidences of gastric cancer and gastric duodenal ulcer are also decreasing. Under such circumstances, the number of reports of AIG, which has thus far been considered rare, is increasing, and it is necessary to revitalize our awareness from an epidemiological point of view. Furthermore, advancements in imaging tests mean that more detailed characteristic findings have been reported, and early imaging findings are being revealed.

Typical AIG has some characteristic findings such as remnants of oxyntic mucosa, hyperplastic polyps, and sticky adherent dense mucus in about 30% of cases, in addition to severe atrophic gastritis. The presence of antral mucosa is not always normal. Early AIG has different characteristics than typical AIG, such as mosaic patterns by reddened and edematous changes in the gastric areas or pseudopolyps.

In IEE observation, it has been suggested that the types of findings change with the progression of AIG inflammation. The findings present in early AIG have been a preserved normal structure. On the other hand, there are some variations in the typical AIG IEE findings. As the AIG stage progresses, the network of subepithelial vessels collapses, and crypt openings become obscured.

The diagnosis of early AIG is important for the management of the clinical and oncological risks of AIG. The further accumulation of cases is needed to reveal the endoscopic features of early AIG. In addition, to enable detailed endoscopic diagnosis, advanced endoscopic techniques, including IEE, are essential.

Endoscopists need to understand the diverse clinical picture of AIG and update their epidemiological awareness and knowledge of characteristic endoscopic findings. Detailed IEE findings and early case findings will contribute to revealing the unexplained natural history of AIG. The accumulation of further cases is expected.

## Figures and Tables

**Table 1:** Summary of previous reports regarding early AIG.

Author	Year	Country	Case No.	Sex	Age	Endoscopic Features	PCA	IFA	Ga. (pg/mL)	PG1 (ng/mL)	PG1/2
Kotera et al. [39]	2020	Japan	1	Male	48	a-1	640×	−	709	unk	unk
			2	Female	70	a-2	640×	+	5265	unk	unk
Ayaki et al. [37]	2021	Japan	1	Female	40	b-1	640×	unk	894	unk	unk
			2	Female	35	b-2	160×	unk	1804	unk	unk
Kishino et al. [38]	2021	Japan	1	Female	50	c	320×	−	820	72.7	4.1
Kotera et al. [40]	2022	Japan	1	Male	66	d	320×	+	338	56.1	7.6

AIG, autoimmune gastritis; PCA, parietal cell antibody; IFA, intrinsic factor antibody; Ga, gastrin; PG, pepsinogen; unk, unknown. a-1: Mild mucosal atrophy and nodules on the greater curvature of the corpus. a-2: Mild mucosal atrophy and nodules on the greater curvature of the corpus. b-1: Polygonal areae gastricae surrounded by a reticular border in a mosaic-like pattern were observed on the greater curvature of the gastric body and fundus. b-2: Non-atrophic mucosa with a mosaic pattern, slight swelling of the areae gastricae, and the presence of erythema in the corpus. c: Reddened and edematous change in the gastric areas, extensively in the gastric fundic gland mucosa without atrophic change. d: Neither atrophic nor inflammatory changes were observed.

## Data Availability

Not applicable.
